# The impact of delayed reporting on forensic evidence recovery in anal sexual assault cases: A retrospective study

**DOI:** 10.1371/journal.pone.0343686

**Published:** 2026-03-18

**Authors:** Erhan Kartal, Yasin Etli, Bedir Korkmaz

**Affiliations:** 1 Head of the Department of Forensic Medicine, Medical Faculty of Van Yuzuncu Yil University, Van, Turkey; 2 Department of Forensic Medicine, Medical Faculty of Van Yuzuncu Yil University, Van, Turkey; 3 Forensic Medicine Specialist, Ministry of Justice, Sivas Forensic Medicine Branch Directorate, Sivas, Turkey; Tarbiat Modares University, IRAN, ISLAMIC REPUBLIC OF

## Abstract

**Background:**

Delayed presentation after sexual assault reduces the probability of detecting injuries or recovering biological evidence. Evidence specific to anal-route allegations and physical findings is comparatively limited.

**Methods:**

We retrospectively reviewed 222 consecutive cases alleging anal-route sexual assault seen at a university forensic clinic in Türkiye (2010–2014). Examinations included standardized external anogenital inspection under magnification (colposcopy) and full-body assessment; anoscopy was not routinely available. The primary outcome was acute physical findings (composite of anal/perianal and, when present, parallel genital injuries, plus extragenital traumatic lesions). Bivariate associations used Pearson’s χ² (with df reported; Monte Carlo where appropriate). As a prespecified sensitivity analysis, a Random Forest classifier was trained using pre-examination demographics and incident-context variables with stratified 5-fold cross-validation, reporting ROC AUC, PR-AUC, accuracy, Brier score, and permutation feature importance.

**Results:**

Most victims were female (69.4%) and <18 years (60.8%). The probability of detecting acute findings was higher with earlier examination (e.g., ≤ 7 days vs. later: OR 6.13, χ² = 88.36, p < 0.001). The Random Forest showed moderate discrimination (CV ROC AUC 0.781 ± 0.077; PR-AUC 0.604 ± 0.119; accuracy 0.739 ± 0.079), with out-of-fold ROC AUC 0.762 (bootstrap 95% CI 0.692–0.827) and Brier 0.186. Assault-to-examination interval was the dominant contributor by permutation importance (group share 43.3%), followed by age metrics, prior anal intercourse count, and contextual factors. Findings were directionally similar when the outcome was any physical finding (genital or extragenital).

**Conclusions:**

Earlier examination markedly increases the likelihood of detecting physical findings in alleged anal sexual assaults. Negative examinations after delays are forensically compatible with the allegation and should be documented with precise timelines and standardized magnified imaging. Streamlined, trauma-informed pathways to facilitate timely reporting may improve evidentiary outcomes and reduce secondary victimization.

## Introduction

Sexual assault is defined as any attempted or completed sexual act committed against a person without their consent, covering a wide spectrum from unwanted sexual contact to rape [1]. International data indicate that more than half of women and men in some populations report lifetime exposure to sexual assault, and that sexual victimization is a major driver of post-traumatic stress and other mental health sequelae [1–4]. Studies from Türkiye report some of the highest prevalence estimates worldwide, underscoring that sexual violence constitutes not only an individual trauma but also a significant public health problem in this context [1,2].

Despite its frequency, sexual assault remains one of the least reported crimes. Population-based and clinical studies consistently show that between two-thirds and nearly all sexual assaults are never reported to the police or legal authorities [5–7]. Even when survivors do disclose, reporting is often delayed: fewer than half present to legal or medical services within the first three days, and a substantial proportion wait months or years before seeking help [8]. Barriers include shame, fear of retaliation, mistrust of authorities, and the expectation that “nothing will be done” [7]. These patterns have typically been framed as a public health and victim-support challenge, but they also have critical implications for forensic practice and criminal justice.

From a medico-legal perspective, the time elapsed between assault and examination is a key determinant of the forensic validity, admissibility, and probative weight of evidence. Biological traces such as spermatozoa and seminal markers diminish rapidly; classical work shows sharp declines in sperm and DNA recovery beyond 24–72 hours, leading many protocols to recommend evidence-collection windows based on time since assault [9,10]. Likewise, genito-anal and extragenital injuries evolve and heal over days to weeks. Multivariable emergency-department studies have demonstrated that shorter post-assault intervals are independently associated with higher odds of detecting anogenital trauma, whereas delayed presentation is associated with lower detection rates [11,12]. At the same time, large series and recent systematic reviews confirm that many survivors—especially those presenting late—have no visible anogenital injury, and that absence of injury does not imply consent or fabrication [13–15].

These clinical realities intersect directly with legal outcomes. In several jurisdictions, the presence of genital or non-genital injury has been associated with increased likelihood of charge filing and conviction, whereas negative examinations may be misinterpreted by investigators, prosecutors, or courts as casting doubt on the allegation [12,16]. Medico-legal guidelines therefore emphasize that (i) medical evidence can rarely resolve the question of consent on its own, (ii) both the presence and absence of injury must be interpreted in light of the assault-to-examination interval, the nature of the acts, and victim characteristics, and (iii) standardized, trauma-informed documentation—including precise timelines—is essential for evidence to be admissible and fairly weighed in court [17–19]. In this sense, delayed presentation is not only a clinical and public health issue; it also shapes the evidentiary landscape on which legal decisions rest, particularly in cases where the forensic medical report is the main objective source of information.

Anal-route sexual assaults occupy a relatively understudied niche within this broader literature. While the time-dependent recovery of spermatozoa and DNA after vaginal assault has been extensively characterized [9,10,20,21], far fewer studies have focused specifically on anal assaults, and even fewer have quantified how delayed presentation affects the likelihood of detecting acute anal or perianal injuries. Yet in many alleged anal assaults, particularly when condom use is reported or seminal fluid is absent, physical findings may constitute the primary remaining source of corroborative forensic evidence. When examinations occur after substantial delay, a normal or negative anogenital exam is medically expected, but may be misused in court to challenge victim credibility unless the time dependence of injury and evidence is explicitly explained.

Türkiye provides a particularly relevant setting for examining these issues. As in many countries, survivors often present first to hospital-based forensic clinics, where standardized documentation and photo-recording may be available, but access to specialized equipment (e.g., anoscopy) can be limited. Forensic physicians are expected to produce detailed reports that may later be scrutinized by prosecutors, defense counsel, and judges. However, empirical data describing how the assault-to-examination interval influences the yield of anal and extragenital findings in this context are scarce, and there is little evidence to guide expert interpretation of negative examinations in delayed anal sexual assault cases.

In this retrospective study, we analyzed 222 consecutive alleged anal sexual assault cases examined at a university forensic clinic in Türkiye. Our primary objective was to quantify how the assault-to-examination interval affects the probability of detecting acute anal/perianal and extragenital findings, using standardized magnified examination. We also conducted a prespecified multivariable sensitivity analysis using a Random Forest classifier to explore the relative contribution of time and contextual factors (e.g., victim age, prior anal intercourse, crime place) to the detection of physical findings. By framing these results explicitly within a medico-legal context, we aim to clarify how delayed presentation should inform the forensic interpretation of both positive and negative examinations in anal sexual assault cases, and to support more evidence-based expert testimony and judicial decision-making.

## Materials and methods

This study was conducted on victims who claimed to have been sexually assaulted and who presented to the Forensic Medicine Department Clinic of Aydın Menderes University Faculty of Medicine in Aydın, Turkey, between 2010 and 2014. The data used in this study were originally collected during a specialty thesis project between June 2015 and December 2015. For the purposes of manuscript preparation and secondary analysis, the anonymized version of the dataset was re-accessed and processed between January 2025 and May 2025. Inclusion criteria were claims of sexual violence via the anal route and genital examinations performed with colposcopy. Exclusion criteria were experiencing sexual assault via routes other than the anal region and not providing consent for the study.

### Ethical considerations

This study was approved by the Non-Interventional Clinical Research Ethics Committee of Aydın Menderes University Faculty of Medicine on 05.05.2015 with decision number 2015/579. Because the research was a retrospective analysis of fully de-identified medical records and posed no more than minimal risk to participants, the Ethics Committee granted a waiver of informed consent in accordance with local regulations and the Declaration of Helsinki. All data were handled in compliance with relevant data-protection legislation, and no personally identifiable information was collected or disclosed.

### Physical examination

Prior to the examination, the procedure was explained, and an informed consent was obtained from each case. A detailed history was then taken, covering aspects such as how and where the incident occurred, the extent of the incident, any prior history of anal intercourse, the object involved in the sexual act, occupations of the victim’s mother and father, and the time elapsed between the incident and the examination. A general body examination was performed to assess external findings related to the sexual assault. This was followed by the anal examination. Anal examinations in adults were conducted in the knee-elbow position, or in the left lateral or supine position for cases where cooperation was not feasible. In pediatric cases, the examination was performed with the child seated on the mother’s lap. In female cases, a vaginal examination for signs of sexual assault was conducted in the lithotomy position. In male cases, the penis and scrotum were also examined for evidence of sexual assault.

During the study period, an anoscope was not available in our clinic. Accordingly, a colposcope was used as an adjunct during the anal and perianal examination. Magnified, well-illuminated visualization facilitated the detection of micro-lacerations, abrasions, and vascular patterns at the anal verge and perianal skin; photo-documentation was obtained when feasible. Prior work indicates that colposcopic magnification can improve the detection and documentation of subtle anogenital trauma, whereas anoscopy is preferable for intra-anal mucosal assessment and targeted evidence collection [22–24]. Our protocol therefore focused on external perianal findings.

In this study, “acute findings” were defined a priori as observable traumatic injuries on physical examination at presentation, consistent with recent assault. This composite outcome included: (i) anogenital lesions—anal/perianal abrasions, edema, ecchymosis, bleeding, and lacerations/tears; in females, parallel genital traumatic findings (e.g., hymenal/labial abrasions or tears, vaginal mucosal lacerations) when present; and (ii) extragenital traumatic findings (e.g., abrasions, contusions, lacerations) temporally attributable to the index event. Findings interpreted as sequelae of prior injury or tissue remodeling (e.g., scars, decreased anal sphincter tone, reflex anal dilatation, asymmetry/indistinctness of anal cushions) were classified as “chronic findings” and analyzed separately. Systemic or non-specific physiological signs (e.g., fever, tachycardia) were not included in the definition of acute findings.

### Statistical analysis

In this study, variables such as age, age group, suspect’s age, suspect’s age group, use of lubricants, the manner in which the sexual assault was carried out, the location of the incident, whether the incident was qualified (qualified incident referred to the aggravated form of sexual assault under the Turkish Penal Code (TCK) Article 102/2, i.e., insertion of a body part or other object into the victim’s body (vaginal, anal, or oral)), the extent of sexual abuse, whether the victim had a history of anal intercourse, the object used during the sexual assault, the time elapsed between the incident and the examination, acute findings of sexual assault via the anal route if present, the location of fissures detected in the anal region, chronic findings of sexual assault via the anal route if present, the location of scars in the anal region, vaginal sexual assault findings, and the occupations of the victims’ parents were retrospectively scanned and recorded. Among these variables, the relationship between the victim’s gender and the location of the incident, the occupations of the victims’ parents and the victim’s gender, the victims’ ages and the location of the incident, and the time elapsed between the incident and the examination date and gender were analyzed. Statistical analyses were performed using SPSS V.23.0. The presence of statistically significant relationships among the analyzed variables was determined using the Pearson Chi-Square test. Monte Carlo Simulation was used when there were insufficient cases within the cells. The level of statistical significance was set at 0.05.

As a prespecified sensitivity analysis, we trained a Random Forest [25] classifier to estimate the probability of acute anal findings and all findings (genital or extragenital) using only pre-examination demographic and incident-context variables. The assault-to-examination interval was encoded as an ordinal predictor; nominal covariates were one-hot encoded with categories fixed a priori. Class imbalance was addressed with class-weighted learning. We quantified discrimination with stratified 5-fold cross-validation, reporting ROC AUC, accuracy, and average precision (PR-AUC) given class imbalance [26]. As an internal check we also reported the model’s out-of-bag (OOB) accuracy and probabilistic metrics (OOB ROC-AUC, PR-AUC, Brier score); because preprocessing is fit on the full dataset, OOB estimates may be mildly optimistic. To provide a single set of probability-based metrics we computed out-of-fold (OOF) predicted probabilities across folds and derived OOF ROC-AUC, PR-AUC, the Brier score, and an optimal classification threshold by Youden’s J [27]; at that threshold we report sensitivity, specificity, PPV, and NPV. For interpretability, we calculated permutation feature importance (mean AUC decrement upon shuffling each feature); importances for multi-level factors were summed across one-hot columns to yield a variable-level importance [28]. To avoid target leakage, direct examination outcomes were excluded from the predictor set. Where indicated, bootstrap resampling (2,000 replicates) was used to obtain a 95% CI for the OOF ROC-AUC. We implemented the analysis in Python (scikit-learn) [29].

## Results

A total of 222 cases were included in the study. Of these, 154 (69.4%) were female and 68 (30.6%) were male. Of the cases, 60.8% (n = 135) were under 18 years old, and 39.2% (n = 87) were over 18 years old. The average age of the cases was 19.88 years (SD: 10.5; median: 17; min: 3; max: 53). The average age for male cases was 17.3 years (SD: 10.2; median: 15; min: 3; max: 49), while for female cases it was 21.0 years (SD: 10.5; median: 18; min: 4; max: 53). There was a statistically significant difference in age distribution between male and female cases (p = 0.002).

All suspects were male, with an average age of 27.2 years (SD: 12.7; median: 24; min: 10; max: 70). In the majority of cases (n = 187; 84.2%), the suspect was 1–55 years older than the victim (p = 0.000). In 16 cases (7.2%), the victim and suspect were the same age, and in 19 cases (8.6%), the victim was 1–17 years older than the suspect.

In the majority of cases (n = 142; 63.9%), no lubrication was used, while saliva was used in 49 cases (22.1%), cream in 20 cases (9%), soap in 5 cases (2.3%), condom in 4 cases (1.8%), and olive oil in 2 cases (0.9%). The most common forms of abuse were physical force (n = 83, 37.3%), deception (n = 47, 21.2%), and threats (n = 27, 12.2%). Numerical data on other forms of abuse are provided in Table 1. When examining the distribution of locations where the abuse occurred, the most common location was a shared house (19.4%), followed by the perpetrator’s residence (18.9%), shared living areas, and open areas (each with 28 cases). It was found that the distribution of locations where the abuse occurred varied significantly according to the victim’s gender (p = 0.000; continuation of Table 2). The most common locations for male victims were shared living areas (25 cases, 36.8%), outdoor areas (14 cases, 20.6%), and rural areas (11 cases, 16.2%), while for female victims, the most common locations were shared houses (42 cases, 27.3%), the perpetrator’s residence (35 cases, 22.7%), and residences of third parties (17 cases, 11%). It was also found that the characteristics of the locations where the incident occurred varied according to whether the victims were over or under 18 years old. For cases under 18 years old, the most common locations were the perpetrator’s residence (28 cases, 23.7%), outdoor areas (23 cases, 19.5%), and rural areas (19 cases, 16.1%), while for cases over 18 years old, the most common locations were shared houses (35 cases, 33.7%), shared living areas (15 cases, 14.4%), and the perpetrator’s residence (14 cases, 13.5%) (Table 2).

**Table 1:** Methods used by perpetrators for sexual abuse.

MODE OF PERPETRATION OF ABUSE		
Mode	N	%
Deception	47	21.2%
Threatening	27	12.2%
Physical Force	83	37.3%
Exploiting Physical/Mental Impairment	12	5.4%
Exploiting Youthfulness	19	8.5%
Physical Force & Threatening	21	9.5%
Physical Force & Exploiting Physical/Mental Impairment	9	4.1%
Threatening & Exploiting Physical/Mental Impairment	4	1.8%
**Total**	**222**	**100.0%**

**Table 2:** Places where sexual abuse occurs and relationship between abuse location and gender.

LOCATION OF THE ABUSE			
Location		N	%
Perpetrator's Residence		42	18,9%
Victim’s Residence		18	8,1%
Residence Belonging to a Third Party		20	9,0%
Social Spaces		4	1,8%
Rural Area		27	12,2%
Inside a Vehicle		7	3,2%
Shared Living Area		28	12,6%
Outdoor Area		28	12,6%
Workplace		5	2,3%
Common House		43	19,4%
**Total**		**222**	**100.0%**
**RELATIONSHIP BETWEEN** **LOCATION OF THE INCIDENT AND GENDER**			
**Location**	**Gender**		**Total (N (%))**
	**Male (N (%))**	**Female (N (%))**	
Perpetrator's Residence	7 (10.3%)	35 (22.7%)	42 (18.9%)
Victim’s Residence	5 (7.4%)	13 (8.4%)	18 (8.1%)
Residence Belonging to a Third Party	3 (4.4%)	17 (11.0%)	20 (9.0%)
Social Spaces	0 (0.0%)	4 (2.6%)	4 (1.8%)
Rural Area	11 (16.2%)	16 (10.4%)	27 (12.20%)
Inside a Vehicle	0 (0.0%)	7 (4.5%)	7 (3.2%)
Shared Living Area	25 (36.8%)	3 (1.9%)	28 (12.6%)
Outdoor Area	14 (20.6%)	14 (9.1%)	28 (12.6%)
Workplace	2 (2.9%)	3 (1.9%)	5 (2.3%)
Common House	1 (1.5%)	42 (27.3%)	43 (19.4%)
**Total**	**68 (100.0%)**	**154 (100.0%)**	**222 (100.0%)**
**RELATIONSHIP BETWEEN** **LOCATION OF THE INCIDENT AND VICTIM AGE**			
**Occupation**	**Age**		**Total (N (%))**
	**Under 18 (N (%))**	**Over 18 (N (%))**	
Perpetrator's Residence	28 (23.7%)	14 (13.5%)	42 (18.9%)
Victim’s Residence	9 (7.6%)	9 (8.7%)	18 (8.1%)
Residence Belonging to a Third Party	11 (9.3%)	9 (8.7%)	20 (9.0%)
Social Spaces	1 (0.8%)	3 (2.9%)	4 (1.8%)
Rural Area	19 (16.1%)	8 (7.7%)	27 (12.2%)
Inside a Vehicle	4 (3.4%)	3 (2.9%)	7 (3.2%)
Shared Living Area	13 (11.0%)	15 (14.4%)	28 (12.6%)
Outdoor Area	23 (19.5%)	5 (4.8%)	28 (12.6%)
Workplace	2 (1.7%)	3 (2.9%)	5 (2.3%)
Common House	8 (6.8%)	35 (33.7%)	43 (19.4%)
Total	118 (100.0%)	104 (100.0%)	222 (100.0%)

*Pearson Chi Square: 79,079; Degree of Freedom (DF): 9; P: 0.000.*

*The null hypothesis stating that there is no relation between gender and incident location was rejected.*

*Pearson Chi Square: 38.630; DF:9; P: 0.000.*

*The null hypothesis stating that there is no relation between age and location of the incident was rejected.*

Of the cases, 187 (84.2%) involved anal penetration, 22 (9.9%) were attempted penetration, and 10 (4.5%) were harassment (Table 3). In 97 cases (43.7%), there was no history of anal intercourse prior to the incident. In 21 cases (9.5%), there was a history of anal intercourse once, and in 104 cases (46.8%), there was a history of anal intercourse two or more times (Table 3).

**Table 3:** The characteristics about the extent of abuse, history of previous anal intercourse, the object used during abuse and the time interval between incident and inspection.

EXTENT OF INCIDENT		
Extent	N	%
Harassment	10	4,5%
Attempt	22	9,9%
Anal Penetration	187	84,2%
Oral	2	0,9%
Attempt & Oral	1	0,5%
**Total**	**222**	**100.0%**
**HISTORY OF ANAL INTERCOURSE BEFORE THE INCIDENT**		
**Anal Intercourse Before the Incident**	**N**	**%**
None	97	43,7%
Once	21	9,5%
Twice	16	7,2%
Three Times	20	9,0%
Four Times	8	3,6%
Five or More Times	60	27,0%
**Total**	**222**	**100,0%**
**OBJECT USED FOR SEXUAL ASSAULT**		
**Object**	**N**	**%**
None	10	4.5%
Penis	185	83.3%
Finger	14	6.3%
Other Objects	7	3.2%
Penis and Finger	4	1.8%
Penis and Other Objects	2	0.9%
**Total**	**222**	**100.0%**
**TIME INTERVAL BETWEEN INCIDENT AND INSPECTION**		
**Time Interval**	**N**	**%**
1 Day	58	26.1%
1-3 Days	21	9.5%
3-7 Days	37	16.7%
7-10 Days	10	4.5%
10 Days – 1 Month	36	16.2%
1 Month-1 Year	48	21.6%
Over 1 Year	12	5.4%
**Total**	**222**	**100.0%**

The time elapsed between the incident and the examination was 1 day in 58 cases (26.1%), 1–3 days in 21 cases (9.5%), and more than 3 days in 143 cases (64.1%) (Table 3). A significant pattern was found when investigating the relationship between the likelihood of detecting acute findings (anogenital or extragenital) and the time elapsed between the incident and the examination. It was found that the likelihood of detecting acute findings was higher in cases presenting within the first 7 days (odds ratio = 6.13; χ2 = 88.36; p = 0.000). A similar comparison between cases presenting within the first 24 hours and those presenting within 1–10 days showed an odds ratio of 1.19 (χ2 = 4.55; p = 0.033), and a comparison between cases presenting within the first 3 days and those presenting after 3 days showed an odds ratio of 3.86 (χ2 = 27.12; p = 0.000). Nevertheless, even among those presenting within the first 24 hours, 29/58 (50.0%) examinations yielded no acute findings; across ≤7 days (1 day + 1–3 days + 3–7 days), negative acute findings were documented in 60/116 (51.7%) cases (Table 4).

**Table 4:** The relationship between the time between the sexual abuse incident and the inspection and the probability of detecting acute findings of anal sexual abuse.

			1 day	1-3 days	3-7 days	7-10 days	10 days- 1 month	1 month-1 year	More than 1 year	Total
**Acute Findings**	**Negative**	**Count**	29	10	21	6	29	45	12	152
		**Percent**	19.1%	6.6%	13.8%	3.9%	19.1%	29.6%	7.9%	100.0%
	**Positive**	**Count**	29	11	16	4	7	3	0	70
		**Percent**	41.4%	15.7%	22.9%	5.7%	10.0%	4.3%	0.0%	100.0%
**Total**		**Count**	58	21	37	10	36	48	12	222
		**Percent**	26.1%	9.5%	16.7%	4.5%	16.2%	21.6%	5.4%	100.0%

When the probability of detecting acute findings in cases admitted in the first 7 days was compared with the probability of detecting acute findings in cases admitted in the following days, the odds ratio was found to be 6.13. (χ2 = 88.36; DF: 1; p = 0.000).

When the probability of detecting acute findings in cases admitted in the first 24 hours was compared with the probability of detecting acute findings in cases admitted between 1–10 days, the odds ratio was found to be 1.19. (χ2 = 4.55; DF: 1; p = 0.033).

When the probability of detecting acute findings in cases admitted in the first 3 days was compared with the probability of detecting acute findings in cases admitted after 3 days, the odds ratio was found to be 3.86. (χ2 = 27.12; DF: 1; p = 0.000).

In the majority of cases (n = 183, 82.4%), no anal fissures were found. Among the cases with fissures, the most common locations were at the 6 o’clock position (n = 11, 28.2%), followed by the 12 o’clock position (n = 8, 20.5%) and the 11 o’clock position (n = 6, 15.4%) (Table 5). Additionally, in the majority of cases (n = 167, 75.2%), no chronic anal sexual assault findings were detected. The most common chronic anal sexual assault findings were scars in 25 cases (11.3%) and asymmetry in the anal cushions in 8 cases (3.6%). The occupations of the victims’ parents were also analyzed. It was found that the majority of the victims’ mothers (n = 122, 55%) were housewives, and their fathers were unskilled workers (n = 55, 24.8%) (Table 5). Considering the average female labor force participation rate in Turkey is 34.5%, a comparison using the labor force participation rate of the mothers of the abused children revealed that children whose mothers were not working or were housewives had a 1.27 times higher risk of sexual abuse (Pearson chi-square 13.878; DF: 1; p = 0.000) [30].

**Table 5:** Characterizations about the anal fissure localizations, chronic anal sexual assault findings, and the occupations of victims’ parents.

LOCATION OF ANAL FISSURES		
Location	N	%
None	183	82.4%
1	4	1.8%
4	1	0.5%
6	11	5.0%
7	4	1.8%
8	2	0.9%
9	3	1.4%
11	6	2.7%
12	8	3.6%
**Total**	**222**	**100.0%**
**CHRONIC ANAL SEXUAL ASSAULT FINDINGS**		
**Findings**	**N**	**%**
None	167	75.2%
Decreased Tonus	2	0.9%
Asymmetry in Anal Cushions	8	3.6%
Indistinctness in Anal Cushions	3	1.4%
Scars	25	11.3%
Reflex Anal Dilatation	3	1.4%
More Than 2 Findings	4	1.8%
Decreased Tonus & Funneling	2	0.9%
Decreased Tonus & Asymmetry in Anal Cushions	4	1.8%
Decreased Tonus & Reflex Anal Dilatation	1	0.5%
Asymmetry in Anal Cushions & Funneling	2	0.9%
Funneling	1	0.5%
**Total**	**222**	**100.0%**
**OCCUPATION OF THE VICTIM'S MOTHER**		
**Occupation**	**N**	**%**
Housewife	122	55.0%
Unemployed	11	5.0%
Farmer	16	7.2%
Unskilled worker	23	10.4%
Officer	3	1.4%
Small Business	10	4.5%
Unknown	29	13.1%
Deceased	7	3.2%
Sex Worker	1	0.5%
**Total**	**222**	**100.0%**
**OCCUPATION OF THE VICTIM'S FATHER**		
**Occupation**	**N**	**%**
Unknown	30	13.5%
Unemployed	15	6.8%
Retired	19	8.6%
Farmer	42	18.9%
Unskilled worker	55	24.8%
Officer	7	3.2%
Small Business	32	14.4%
Deceased	22	9.9%
**Total**	**222**	**100.0%**

The null hypothesis that the lesions were equally distributed across all clockwise localizations was rejected. (Pearson chi-square: 43.154; DF: 8; p = 0.000).

Considering that the average labor force participation rate for women in Turkey is 34.5% (30) a comparison using the labor force participation rate of mothers of abused children reveals that children whose mothers are unemployed or homemakers have a 1.27 times higher risk of experiencing sexual abuse (Pearson Chi-square 13.878; DF:1; p = 0.000).

Random Forest performance was moderate on stratified 5-fold cross-validation (ROC AUC 0.781 ± 0.077; PR-AUC 0.604 ± 0.119; accuracy 0.739 ± 0.079). Out-of-fold (OOF) probabilities yielded a comparable single-set estimate (ROC AUC 0.762, bootstrap 95% CI 0.692–0.827; PR-AUC 0.549; Brier score 0.186). At the Youden J–maximizing threshold (0.426), sensitivity was 0.729, specificity 0.697, positive predictive value (PPV) 0.526, and negative predictive value (NPV) 0.848. Out-of-bag (OOB) internal validation was similar (OOB accuracy 0.734; OOB ROC AUC 0.734; OOB PR-AUC 0.534; OOB Brier 0.192). By permutation feature importance, the assault-to-examination interval was the dominant contributor (group share 43.3%), followed by age-related variables (victim-perpetrator age difference, victim age, perpetrator age), prior anal intercourse count and contextual categories for crime place and assault extent (e.g., crime place total 9.1%, extend of assault total 4.2%). Feature importances are provided in Table 6.

**Table 6:** Permutation feature importance (Random Forest) for predicting (A) acute anal findings and (B) any findings (genital or extragenital).

Random Forest Permutation Importance form Predicting Acute Anal Findings		
Variable group	Importance (sum)	Share (%)
**Assault-to-examination interval**	0.1650	43.3
**Crime Place**	0.0347	9.1
**Victim-Perpetrator Age Difference**	0.0309	8.1
**Victim Age**	0.0290	7.6
**Prior Anal Intercourse Count**	0.0286	7.5
**Perpetrator Age**	0.0250	6.5
**Extent of Assault**	0.0161	4.2
**Victim Marital Status**	0.0098	2.6
**Lubrication Used or Not**	0.0080	2.1
**Force Used or Not**	0.0073	1.9
**Random Forest Permutation Importance for Predicting All Findings (Genital or Extragenital)**		
**Variable group**	**Importance (sum)**	**Share (%)**
**Assault-to-examination interval**	0.0580	22.1
**Victim Age**	0.0460	17.6
**Prior Anal Intercourse Count**	0.0320	12.2
**Victim-Perpetrator Age Difference**	0.0210	8.0
**Perpetrator Age**	0.0200	7.5
**Force Used or Not**	0.0160	6.2
**Crime Place**	0.0160	6.1
**Victim Sex**	0.0120	4.6
**Lubrication Used or Not**	0.0090	3.4
**Extent of Assault**	0.0066	2.5
Importance = mean decrease in ROC AUC from permutation. Share (%) sums to 100 within each outcome. Grouped importance is the sum across one-hot levels for the same variable.		

We repeated the prespecified Random Forest sensitivity analysis using a binary outcome of any physical finding (genital or extragenital). Discrimination was moderate on stratified 5-fold cross-validation (ROC AUC 0.767 ± 0.033; PR-AUC 0.887 ± 0.027; accuracy 0.739 ± 0.028). Out-of-fold (OOF) probabilities yielded similar performance (ROC AUC 0.770, bootstrap 95% CI 0.700–0.838; PR-AUC 0.875; Brier score 0.174). At the Youden J–maximizing threshold (0.581), sensitivity was 0.701, specificity 0.738, positive predictive value (PPV) 0.866, and negative predictive value (NPV) 0.505. Out-of-bag (OOB) internal validation was comparable (OOB accuracy 0.743; OOB ROC AUC 0.766; OOB PR-AUC 0.879; OOB Brier 0.174). Permutation feature importance indicated that the assault-to-examination interval remained the most influential variable (group share 22.1%), followed by victim age (17.6%), prior anal intercourse count (12.2%), victim perpetrator age difference (8.0%), perpetrator age (7.5%), force used (6.2%), crime place categories (6.1%), and victim sex (4.6%). Feature importances are summarized in Table 6. As permutation importance reflects the decrease in AUC upon shuffling, values quantify relative contribution, not directionality or causality.

Across both models, the assault-to-examination interval was the dominant contributor to prediction, which is consistent with our a priori hypothesis that delayed presentation decreases the likelihood of detecting acute anal injury and any medicolegal findings. The ranking of secondary contributors (e.g., age-related variables and, to a lesser extent, crime place and assault extent) provides a data-driven view of contextual factors that may influence evidence yield.

## Discussion

Sexual assaults are among the most serious crimes committed against a person and have serious consequences for both the victim and the suspect [31]. The fact that reported lifetime sexual assault prevalence rates for some groups exceed 50% indicates that this crime occurs at an alarmingly high frequency [1–4,32]. However, despite its severity and prevalence, it has been determined that only very small percentages of sexual assaults are reported to authorities [5–7]. Furthermore, the majority of individuals who do report cases do so with significant delays [8]. This highlights that delayed reporting or non-reporting of sexual assaults is a critical issue requiring urgent attention and resolution. This study was conducted to emphasize this problem.

Although it is empirically known that delayed reporting in sexual assault cases reduces the likelihood of obtaining evidence, much of the time-to-evidence literature indeed concerns biological markers (e.g., spermatozoa, PSA), yet a smaller body of multivariable work has examined physical injury detection, generally finding lower odds with longer assault-to-examination intervals [11–13]. A review of the literature reveals that nearly all studies investigating the relationship between assault-to-examination interval and evidence recovery in sexual assault cases have focused primarily on spermatozoa, DNA, or prostatic acid phosphatase–prostate specific antigen (AP–PSA/p30). In a study conducted by Siriraj, it was found that the rate of sperm detection in sexual assault cases presenting within the first 24 hours was 85%, while the rate of obtaining a DNA profile was 70%. These rates decreased to 55% and 40%, respectively, for the 24–72-hour period, and to 30% and 20% for cases presenting after 72 hours. These findings underscore the critical importance of the first 24 hours and the 72-hour period in terms of evidence collection [16]. Based on the analysis of 5,581 swabs collected from approximately 1,450 sexual assault cases, Casey et al. demonstrated a marked decline in the detection rates of sperm in vaginal and anal swabs after 48 hours post-assault, and in oral swabs beyond 15 hours, thereby emphasizing the critical impact of delayed reporting on the availability of biological evidence [9].

A literature review also summarized the findings of 14 studies that analyzed the maximum persistence times of spermatozoa in the vaginal cavity. These studies reported maximum persistence durations ranging from 14 hours to 7 days, with a mean of 111.3 hours and a median of 132 hours. In the same review, the maximum persistence times of prostatic acid phosphatase (AP) and prostate-specific antigen (PSA/p30) in the post-coital vaginal cavity were also summarized based on data from multiple studies. The reported maximum persistence of AP ranged from 14 hours to 3 days, with most values falling between 24 and 72 hours. For PSA/p30, the maximum persistence ranged from 16 hours to over 72 hours, although several studies noted that PSA levels at longer time points may fall within endogenous ranges, complicating interpretation [20]. Although some advanced techniques have been developed to improve the likelihood of recovering spermatozoa, and a certain degree of success has been reported, the probability of detecting spermatozoa 3–4 days after the incident has been reported to be approximately 32% [21]. Although our study did not include an analysis of sperm, spermatozoa, AP, or PSA, the findings summarized above underscore the critical importance of early reporting in maximizing the likelihood of evidence recovery. Importantly, negative examinations also occurred in early presenters: within 24 hours, 29/58 (50.0%) had no acute findings, and within ≤7 days, 60/116 (51.7%) were negative (Table 4), underscoring that a normal early exam does not exclude assault.

Our sensitivity analyses using Random Forests identified the assault-to-examination interval as the dominant predictor of (A) acute anal findings and (B) any physical finding, with age-related variables and certain contextual factors contributing more modestly. These patterns are consistent with multivariable studies from emergency-department cohorts: Sachs and Chu reported that a post-coital interval <24 hours independently increased the odds of genito-rectal injury (adjusted OR≈7.5) alongside rectal penetration and resistance behaviors, in a logistic-regression model controlling for key covariates [12]. Likewise, Drocton et al. examined consecutive female survivors and found that time from assault to exam (among other assault characteristics) correlated with anogenital injury in multivariable analysis [11]. Large cross-sectional series further demonstrate that no visible anogenital injury is common after sexual assault -even in the absence of prior intercourse- underscoring that a normal exam does not exclude assault [14]. In South Africa, Jina et al. modeled factors associated with the absence of genito-anal injury; although the primary predictors differed (e.g., virginity status, multiple perpetrators, examiner qualifications), the work reinforces how clinical and contextual variables shape injury documentation [15]. Complementing the physical-findings literature, forensic biology studies show steep time-dependent declines in sperm/seminal markers and inform recommended evidence-collection windows -again highlighting the probative premium on early presentation [13]. Taken together, our ML-based importance rankings align with prior multivariable evidence that shorter assault-to-exam intervals most strongly favor detection, while also offering a transparent, data-driven ordering of secondary factors specific to anal-route allegations.

Because permutation importance reflects the drop in AUC when a feature is shuffled, it quantifies predictive contribution rather than causation. These results should therefore be read alongside classical estimates (e.g., odds ratios) reported in prior regression studies and with the acknowledgement that many clinically genuine assaults will have no visible injury, particularly after delays.

Although few studies have isolated the time-dependent probability of specific contusion (bruise) detection, several multivariable analyses have linked shorter post-coital intervals with higher odds of genito-anal injury detection overall [11,12]. It is classically recognized that bruises typically resolve within 10–14 days, gradually changing in color from red to purple, then to green and yellow before disappearing [33]. However, some studies have reported that this resolution may occur within a week or even just a few days in certain cases [34]. Similarly, it is known that simple abrasions typically heal within two weeks at most [35], while more severe lacerations generally resolve within four to six weeks [36]. In light of the summarized literature findings and the data obtained in our study, the critical importance of early reporting in sexual assault cases becomes clearly evident. To reduce delays, it may be possible to implement educational initiatives targeting at-risk populations and establish dedicated support services to encourage timely reporting and access to forensic evaluation. Considering that a significant number of sexual assault victims report delaying their presentation due to fear or reluctance to involve the police, the establishment of specialized support units—such as those implemented in Denmark [37]- could offer meaningful contributions toward addressing this issue. We believe that by exploring the root causes of delayed reporting identified in the literature, it may be possible to develop more targeted and effective interventions.

Beyond logistical constraints, psychological and sociocultural factors in Türkiye also contribute to delayed presentation. Official guidance and legal scholarship highlight the risk of secondary victimization during investigative and judicial procedures, which can deter victims from seeking help or engaging with the system [38,39]. Population-based work in Türkiye documents the persistence of rape myths and victim-blaming attitudes—for example, the tendency to view date rape as less serious than stranger rape—which may undermine perceived legitimacy of complaints and discourage prompt disclosure [40,41]. Psychometric data further show that rape-myth acceptance is measurable in Turkish samples, underscoring the need for training of police, judiciary, and healthcare professionals [42]. In this context, expansion of trauma-informed practice and the broader use of forensic interview rooms (Adli Görüşme Odaları) have been recommended to reduce secondary victimization and facilitate earlier reporting [38,39].

In anal sexual-assault cases, a normal or negative anogenital examination after a delay is medically expected and does not exclude the reported assault. Adolescent data show that genital/body injuries are not routinely present even when assault is alleged, so the absence of injury should not be interpreted as evidence of consent or fabrication [43]. In court, such negative findings can be misused; therefore, expert testimony should explain the time-dependence of injury and biological evidence and make clear that many examinations yield no visible trauma after delays [17]. Consistent with forensic guidelines, medico-legal documentation should (i) use objective, qualified language (e.g., “no acute findings on examination,” not “no evidence of assault”), (ii) photo-document pertinent negative and positive areas, (iii) record precise timelines (assault-to-exam interval, condom use, lubricants) that may explain absent semen or injury, and (iv) note any limitations (e.g., lack of anoscopy for intranal mucosa) [18]. These practices align with ACOG and federal protocol recommendations for standardized, trauma-informed medical-forensic care and courtroom testimony [19]. Finally, protocols emphasize that even when evidence collection windows have lapsed, an exam remains appropriate for medical care and documentation -again underscoring why a negative exam after delay is forensically compatible with the allegation [18].

Parental unemployment is a well-established risk factor for child maltreatment. In a meta-analysis of 60 studies, paternal or any parental unemployment was found to be associated with a 29% increased risk of sexual abuse, a 54% increased risk of neglect, and a 60% increased risk of physical abuse [44]. Another study found that maternal unemployment was a significant risk factor associated with the severity of injury following child abuse [45]. In our study, analysis revealed that children whose mothers were not employed had a 27% higher likelihood of experiencing sexual abuse, which appears to be consistent with the rates reported in the literature. In light of these findings, it is suggested that educating parents -particularly those in households where the mother is unemployed or a homemaker- may be beneficial in reducing the risk of abuse and enhancing protective measures for children.

Our sample comprises only individuals who presented to a university forensic clinic and met our inclusion criteria during 2010–2014 (i.e., anal-route allegations, examined with colposcopy, and eligible records). Although we included all consecutive eligible cases during the study window, this reduces selection within the clinic but does not equate to random sampling of all assaults. Individuals who never seek care -or who present to non-hospital settings- may differ systematically in demographics, reporting delays, severity or visibility of injury, and willingness to undergo examination. If persons with more visible injuries are more likely to present early, our estimates could overstate the strength of the association between shorter delays and detecting acute findings; conversely, if lack of anoscopy led us to miss some intra-anal lesions among presenters, the absolute prevalence of acute findings may be underestimated. Overall, results should be generalized with caution to hospital-presenting cases rather than to all anal sexual assaults in the community.

## Conclusion

In this retrospective cohort of alleged anal-route sexual assaults, the likelihood of detecting acute anogenital and extragenital findings decreased substantially with longer assault-to-examination intervals. At the same time, a normal/negative examination remains possible even with prompt evaluation and should not be interpreted as evidence against the allegation. Classical bivariate results and an exploratory Random Forest analysis were concordant in identifying the time interval as the dominant determinant of detection, with age-related variables and selected contextual factors contributing more modestly. Clinically and forensically, these findings underscore the need for timely presentation, standardized magnified documentation, and careful contextualization of negative examinations after delays, which remain medically expected and do not exclude the allegation. System-level measures that facilitate early reporting and reduce secondary victimization may improve evidentiary yield. Generalizability is limited to hospital-presenting cases, and the absence of routine anoscopy may underestimate intra-anal injuries.

## Supporting information

S1 Data De-identified study dataset. This file contains the case-level variables necessary to reproduce the statistical results presented in this study.
(CSV)
